# Reliability of Tracheal Temperature as a Measurement of Core Body Temperature During Cardiac Surgery Using Cardiopulmonary Bypass

**DOI:** 10.3390/jcm14020632

**Published:** 2025-01-19

**Authors:** Hyun-Uk Kang, Sou-Hyun Lee, Ji-Hyun Chin, In-Cheol Choi, Kyungmi Kim

**Affiliations:** 1 Department of Anesthesiology and Pain Medicine, Asan Medical Center, University of Ulsan College of Medicine, Seoul 05505, Republic of Korea; hyunuk_kang@naver.com (H.-U.K.); cjh@amc.seoul.kr (J.-H.C.); icchoi@amc.seoul.kr (I.-C.C.); 2 Department of Anesthesiology and Pain Medicine, Kyungpook National University Hospital, Daegu 41944, Republic of Korea; youlion6@gmail.com; 3 Department of Anesthesiology and Pain Medicine, Anam Hospital, Korea University College of Medicine, Seoul 02841, Republic of Korea

**Keywords:** body temperature, monitoring, cardiac surgery, cardiopulmonary bypass

## Abstract

**Background**: To compare tracheal temperature (T_ET_) with nasopharyngeal temperature (T_NP_) in patients undergoing cardiac surgery using cardiopulmonary bypass (CPB). **Methods**: T_ET_ was measured using a thermistor in the cuff of an endotracheal tube and T_NP_ was monitored using an esophageal stethoscope. Depending on the management of the CPB strategy, the operation was divided into four periods (pre-CPB, cooling, rewarming, and post-CPB). A Bland–Altman analysis was carried out to compare T_ET_ with T_NP_ in each period. The concordance correlation coefficient for repeated measures analysis and various time lags was used to validate the time lag maximizing the concordance correlation coefficient between the two methods. **Results**: A total of 7191 pairs of temperature recordings acquired from 24 patients were included in the analysis. During steady normothermia, 81.7% (95% confidence interval [CI], 79.8–83.5%) of the pairs had a difference within ±0.5 °C, with a mean bias of −0.28 °C and limits of agreement (LOA) of −0.74 °C to 0.18 °C. The LOA during the cooling and rewarming phase of CPB were −1.13 °C to 0.51 °C and −0.91 °C to 1.29 °C, respectively. The mean bias and LOA throughout the entire operation were −0.10 °C and −0.98 °C to 0.77 °C, respectively. Throughout the entire operation, −2 min of time lag on T_ET_ maximized the concordance correlation coefficient (0.94 [95% CI, 0.92–0.96] to 0.95 [95% CI, 0.93–0.96]), indicating an earlier response of T_ET_ than T_NP_. **Conclusions**: T_ET_ could be an alternative to T_NP_ during cardiac surgery using CPB.

## 1. Introduction

Thermoregulation under general anesthesia depends on autonomic defenses and the external environment because unconsciousness and paralysis incapacitate behavioral regulation [1]. Unintended intraoperative hypothermia can result in adverse consequences such as impaired coagulation and platelet dysfunction, wound infection, or poor early outcomes including increased length of stay in the intensive care unit and hospital as well as mortality [2,3]. Accordingly, the American Society of Anesthesiologists standards for basic anesthetic monitoring recommends observing the body temperature in all patients receiving anesthesia when clinically significant changes in body temperature are intended, anticipated, or suspected.

However, in cardiac surgeries involving cardiopulmonary bypass (CPB), hypothermia is used as a part of the neuroprotective strategy [4]. Since rapid cooling and rapid rewarming might, respectively, result in the formation of gaseous emboli and outgassing, the importance of body temperature monitoring is even more highlighted during the cooling and rewarming phases of CPB [4]. Pulmonary artery or nasopharyngeal temperature (T_NP_) monitoring, which reflects the core body temperature, is, therefore, recommended during the weaning and post-bypass periods [5]. However, pulmonary artery catheterization tends to be complicated [6] and is being used less commonly in cardiac surgery [7]. Also, the reliability of T_NP_ could be influenced by the depth of the probe [8] or hemodynamic status [9].

Previous studies reported that the cuff temperature of an endotracheal tube was a valid core body temperature in intubated patients [10,11]. However, these studies compared the tracheal temperature (T_ET_) with blood temperature and not T_NP_. We hypothesized that the body temperature measured with a thermistor embedded in the balloon cuff of an endotracheal tube could be an alternative to T_NP_ during cardiac surgery using CPB. Therefore, in this study, we compared T_ET_ measured from an endotracheal tube with T_NP_ during steady normothermia, during rapid temperature change, and throughout cardiac surgery using CPB.

## 2. Materials and Methods

### 2.1. Study Design and Participants

This observational cohort study was conducted at Asan Medical Center, a tertiary care center in Seoul, South Korea. Consecutive patients who underwent elective cardiac surgery using CPB with mild hypothermia (32–34 °C) [5] were included from July 2021 to August 2021. We excluded patients who were (1) scheduled for emergency surgery, (2) planned for one-lung ventilation during the operation, or (3) planned for a target temperature lower than 32 °C.

This study was performed according to the principles of the Declaration of Helsinki and was approved by the Institutional Review Board of Asan Medical Center (AMC IRB 2021-0606). All the participants provided written informed consent. This study is registered in the Korean Clinical Trials Registry (KCT 0006345). This study was performed in accordance with Strengthening the Reporting of Observational Studies in Epidemiology (STROBE) criteria [12].

### 2.2. Clinical Data

We analyzed the following baseline characteristics: age, sex, disease history (i.e., diabetes mellitus, hypertension, cerebrovascular accident, peripheral vascular disease, chronic obstructive pulmonary disease, chronic kidney disease with or without renal replacement therapy, and prior cardiac disease), primary percutaneous coronary intervention, coronary artery bypass grafting, myocardial infarction, and congestive heart failure. The European System for Cardiac Operative Risk Evaluation II, aortic cross-clamp duration, and total operation time were also recorded. All the clinical data were extracted from the Asan Medical Center Cardiovascular Surgery and Anesthesia Database and from a retrospective review of the computerized patient record system.

### 2.3. Intraoperative Temperature Monitoring and Management

**Figure 1:**
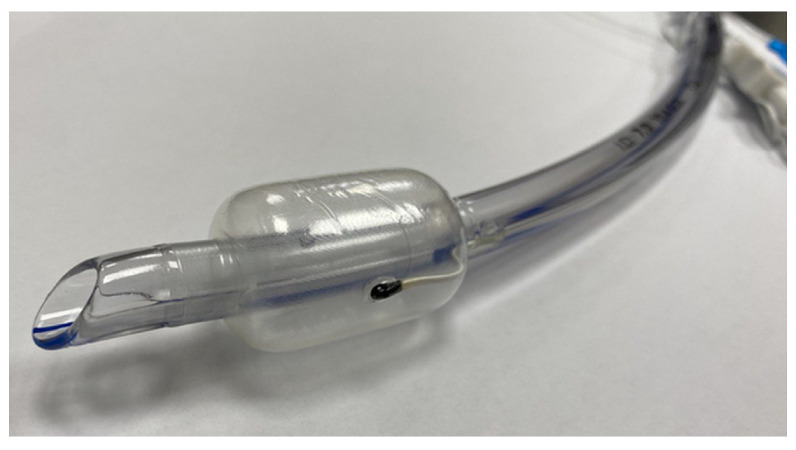
Human endo tube. A thermistor is embedded between the double-layered balloon cuff of an endotracheal tube. When the cuff is inflated, the thermistor is kept attached to the tracheal mucosa [13].

During anesthetic induction, patients were intubated with a single-lumen endotracheal tube that has a thermistor between the double-layer of the balloon cuff (Human Endo Tube, INSUNG Medical, Wonju-si, Republic of Korea; Figure 1). Then, a conventional esophageal stethoscope with a temperature sensor was positioned in the nasopharynx for T_NP_ monitoring. After the induction of anesthesia, both T_ET_ and T_NP_ were recorded minute-by-minute in the electronic medical records system at our institution.

The intraoperative body temperature of the patients was managed during CPB by cardiac surgeons according to their preference. Intraoperative temperature management strategies were as follows: After the initiation of CPB, T_NP_ passively fell to 32 °C, and no active cooling was allowed during CPB. Once T_NP_ fell below 32 °C, the perfusionists rewarmed the blood to maintain the oxygenator arterial outlet blood temperature at 32–34 °C. The operators usually ordered rewarming at some point before removing the aortic cross-clamp, and CPB was terminated after the T_NP_ reached 35–35.5 °C.

### 2.4. Statistical Analysis

Continuous variables are expressed as mean ± standard deviation (SD) or median with interquartile range as appropriate. Categorical variables are expressed as numbers with percentages.

In a previous study that compared T_ET_ with T_NP_, the mean bias between the tracheal and esophageal temperature was −0.22 °C, and the between-subject SD of the mean bias between the tracheal and esophageal temperature was 0.11 °C [11]. Assuming a type I error of 0.05, a type II error of 0.1, and a maximum allowed difference of 0.45 °C [11], the minimum required number of pairs was 1795. Using minute-by-minute intraoperative temperature pairs with an anticipated drop rate of 10%, 34 patients were required for analysis.

To determine the correlation between T_ET_ and T_NP_ in the steady normothermic condition, we compared T_ET_ with T_NP_ before and after CPB when the T_NP_ was 36 °C or greater. The mean bias, SD of the mean bias, and the upper and lower limits of agreement (LOA) of these pairs of recordings were calculated using a Bland–Altman analysis [14]. As a measure of accuracy, the proportion of the pairs whose difference was within 0.5 °C was calculated. The 95% confidence interval (CI) of the proportion was calculated by Wilson’s method [15].

To determine the reliability of T_ET_ in circumstances with rapid temperature changes, the temperature recordings of each participant were divided into four periods: pre-CPB (preparation period for the initiation of CPB), cooling (from the initiation of CPB to the time of the lowest T_NP_ detected), rewarming (from the time of the lowest T_NP_ measured to the termination of CPB), and post-CPB (from the termination of CPB to the end of the operation). The Bland–Altman analysis was used to calculate the mean bias, SD of the mean bias, and the upper and lower LOA in each period, and the proportion of pairs whose difference was within 0.5 °C was analyzed in each period as well. The concordance correlation coefficient for repeated measures [16] was also analyzed, and various time lags were applied to the tracheal temperature to determine the time lag that maximized the concordance correlation coefficient between the two methods. All the statistical analyses were performed using R version 4.0.4 (R Foundation for Statistical Computing, Vienna, Austria).

## 3. Results

### 3.1. Participants

**Figure 2:**
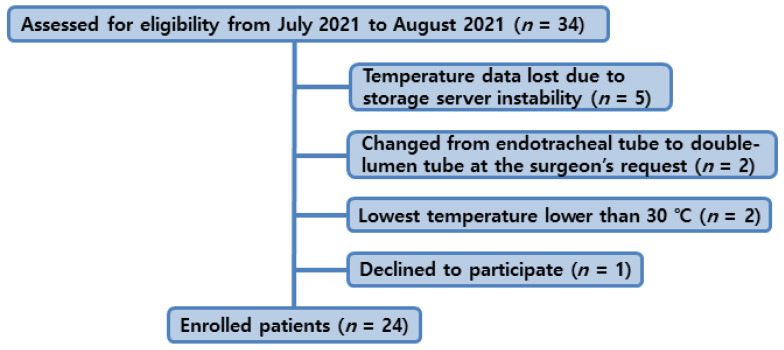
Flow chart of study population.

Of a total of 34 patients who underwent elective cardiac surgery using CPB with mild hypothermia during the study period, 10 dropped out (temperature data lost due to storage server instability, *n* = 5; changed from endotracheal tube to double-lumen tube at the surgeon’s request, *n* = 2; lowest temperature lower than 30 °C, *n* = 2; withdrew consent, *n* = 1). A total of 24 patients (median age, 65.5 years) were included in the final analysis (Figure 2, Table 1). The mean duration of the aortic cross-clamp and operation was 80.5 ± 36.3 and 261.4 ± 77.5 min, respectively.

### 3.2. Temperature Monitoring During Steady Normothermia

**Figure 3:**
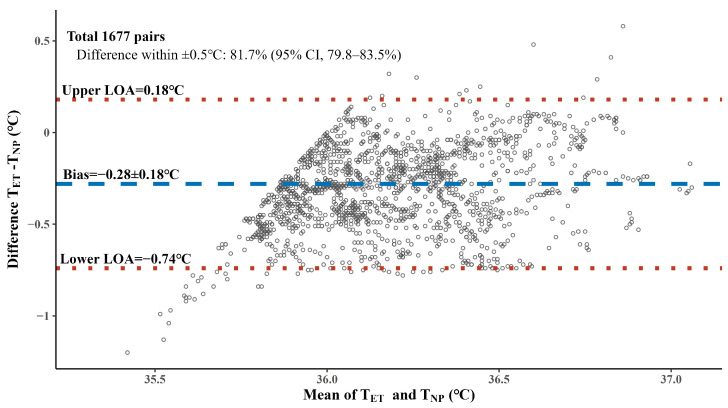
Bland–Altman analysis for tracheal temperature versus nasopharyngeal temperature during steady normothermia. The horizontal dashed line (blue) indicates the mean bias. The horizontal dotted lines (red) indicate the levels of agreement. T_ET_, tracheal temperature; T_NP_, nasopharyngeal temperature; CI, confidence interval; LOA, limit of agreement.

The number of pairs of temperature data from the pre-CPB period and the post-CPB period was 3671, including 1677 pairs whose T_NP_ was ≥36.0 °C. In the Bland–Altman analysis of the temperature data acquired during steady normothermia, the mean bias and SD of the mean bias were −0.28 °C and 0.18 °C, respectively (Figure 3). The LOA during steady normothermia was 0.74 °C to 0.18 °C. Among the 1677 pairs, 1370 pairs (81.7% [95% CI, 79.8–83.5%]) had differences within 0.5 °C.

### 3.3. Temperature Monitoring During the Classified CPB Periods

**Figure 4:**
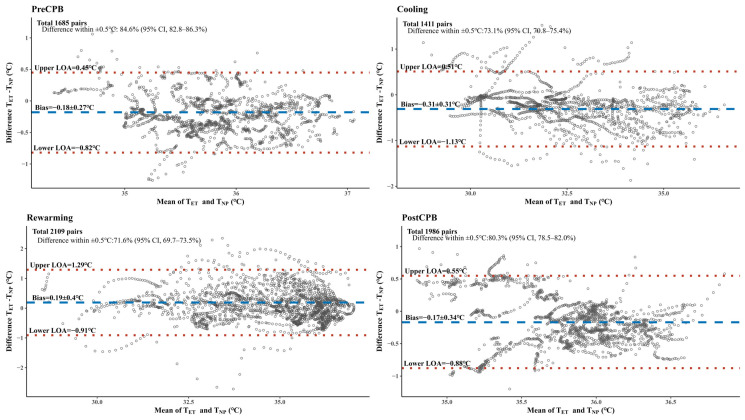
Bland–Altman analyses for each period of cardiopulmonary bypass. The horizontal dashed lines (blue) indicate the mean bias. The horizontal dotted lines (red) indicate the levels of agreement. CPB, cardiopulmonary bypass; T_ET_, tracheal temperature; T, nasopharyngeal temperature; CI, confidence interval; LOA, limit of agreement; Pre-CPB, preparation period for the initiation of CPB; cooling, from the initiation of CPB to the time of the detection of the lowest nasopharyngeal temperature; rewarming, from the time of the detection of the lowest nasopharyngeal temperature to the termination of CPB; Post-CPB, from the termination of CPB to the end of the operation.

A total of 1685, 1411, 2109, and 1986 pairs were recorded during the pre-CPB, cooling, rewarming, and post-CPB periods, respectively. The results of the Bland–Altman analyses for each period are summarized in Figure 4. The mean (SD) bias of each period was −0.18 (0.27) °C, −0.31 (0.31) °C, 0.19 (0.4) °C, and –0.17 (0.34) °C, respectively. The LOA of each period was −0.82 to 0.45 °C, −1.13 to 0.51 °C, −0.91 to 1.29 °C, and −0.88 to 0.55 °C, respectively. Among the total recording pairs of each period, 84.6% (95% CI, 82.8–86.3%), 73.1% (95% CI, 70.8–75.4%), 71.6% (95% CI, 69.7–73.5%), and 80.3% (95% CI, 78.5–82.0%) pairs had a difference within 0.5 °C, respectively.

The results of time lag analyses are summarized in Figure S2. In the pre-CPB period, −1 min of time lag on T_ET_ maximized the concordance correlation coefficient (95% CI) for repeated measures (0.78 [0.63–0.88] to 0.79 [0.63–0.88]). In the cooling period, −2 min of time lag on T_ET_ maximized the concordance correlation coefficient (95% CI) for repeated measures (0.88 [0.63–0.88] to 0.90 [0.84–0.94]). In the rewarming period, −2 min of time lag on T_ET_ maximized the concordance correlation coefficient (95% CI) for repeated measures (0.95 [0.92–0.97] to 0.96 [0.94–0.98]). Finally, in the post-CPB period, −3 min of time lag on T_ET_ maximized the concordance correlation coefficient (95% CI) for repeated measures (0.61 [0.39–0.76] to 0.62 [0.34–0.80]).

### 3.4. Temperature Monitoring Throughout the Entire Operation Period

**Figure 5:**
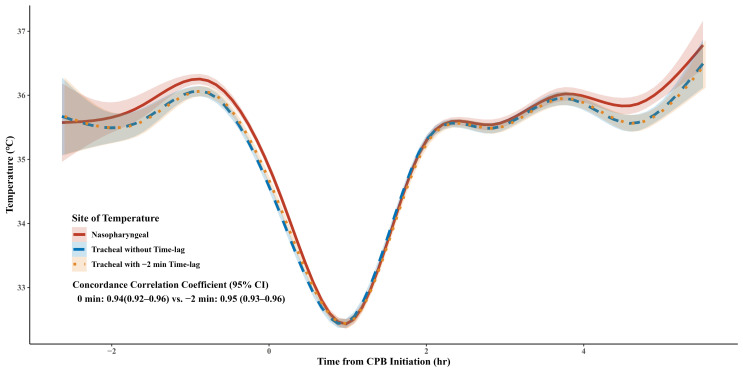
Tracheal and nasopharyngeal temperatures throughout the entire operation period. CI, confidence interval.

A total of 7191 pairs of temperature recordings were acquired. Changes in T_ET_ and T_NP_ throughout the operation are depicted in Figure 5. In the Bland–Altman analysis, the mean bias and SD of mean bias of T_ET_ compared with T_NP_ was −0.10 °C and 0.22 °C, respectively, and the LOA was −0.98 °C to 0.77 °C (Figure S1). Among the 7191 pairs, 77.4% (95% CI, 76.4–78.3%) pairs had a difference within 0.5 °C. The concordance correlation coefficient for repeated measures was 0.94 (95% CI, 0.92–0.96), which was maximized by −2 min of time lag on T_ET_ (0.95 [95% CI, 0.93–0.96]; Figure 5 and Figure S2).

## 4. Discussion

In this study, we compared T_ET_, which was measured using a thermistor embedded within the balloon cuff of an endotracheal tube, with T_NP_, which was measured by an esophageal stethoscope temperature sensor. During steady normothermia, T_ET_ was well correlated with the T_NP_; during rapid temperature changes, however, the differences between T_ET_ and T_NP_ increased. The results of the time lag analyses indicated that T_ET_ had more rapid changes than T_NP_. Although these time lags showed differences between T_ET_ and T_NP_ at the same time, there was a close correlation between T_ET_ and T_NP_ with –2 min of time lag on T_ET_ throughout the entire operation period.

The accuracy and reliability of T_ET_ as a measure of core temperature were demonstrated in previous studies. Yamakage and colleagues [10] compared T_ET_ with temperature recordings from the urinary bladder, rectum, forehead, jugular vein, and arterial outlet of CPB in patients undergoing cardiac surgery using CPB. They used an endotracheal tube with a thermistor attached to the anterior inner surface of the balloon cuff, and showed that blood temperature from CPB, jugular vein temperature, and T_ET_ showed nearly identical time courses throughout the surgery. Also, T_ET_ had good correlation coefficients with both the blood temperature from CPB (*r* = 0.993, *p* < 0.001) and the jugular vein temperature (*r* = 0.993, *p* < 0.001).

In another study, Haugk and colleagues [11] used an endotracheal tube with a double-layer, high-volume, low-pressure balloon cuff with a flat temperature sensor embedded between the two cuff layers to compare T_ET_ with esophageal temperature and pulmonary arterial blood temperature in patients undergoing mild therapeutic hypothermia after cardiac arrest. Consistent with the findings of our present study, they observed a very small hysteresis and good agreement between the tracheal and pulmonary arterial blood temperatures. Also, they concluded that T_ET_ was an accurate and reliable measure of core body temperature when compared with esophageal (mean bias, −0.22 °C; SD of the mean bias, 0.11 °C; LOA, −0.49 °C to 0.07 °C) and pulmonary arterial blood temperatures (mean bias, −0.16 °C; SD of the mean bias, 0.07 °C; LOA, −0.36 °C to 0.04 °C). In contrast, our study compared T_ET_ with T_NP_ only and not with the blood temperature or distal esophageal temperature; accordingly, the distinction of reference value might show inconsistent results with previous studies.

Although T_NP_ is preferred for core temperature measurement in cardiac surgery, it is limited in representing the cerebral temperature. Johnson and colleagues [17] reported a poor correlation between T_NP_ and the blood temperature from the arterial outlet of CPB during the rewarming phase of CPB. Kaukuntia and colleagues [18] found that T_NP_ lagged behind the jugular bulb venous temperature during both the cooling and rewarming phases during CPB. Nussmeier and colleagues [19] also found that T_NP_ overestimated the jugular bulb venous temperature during the cooling phase and underestimated the jugular bulb venous temperature during the rewarming phase of CPB. In agreement with our study, these studies indicated that during rapid temperature changes, T_NP_ has some delay in reflecting the core body temperature. Therefore, a well-designed endotracheal tube with a thermistor may be a feasible monitoring tool to replace the nasopharyngeal stethoscope. Further experimental studies are needed.

### Limitations

Our study has several limitations. Before the final analysis, a considerable number of patients dropped out because of various reasons such as the withdrawal of consent, change in anesthetic or operative plan, and incomplete data. However, we acquired a sufficient number of pairs of temperature recordings from the participants included in the final analysis. Thus, the statistical power remained significant. Also, although we observed an earlier response with T_ET_ than T_NP_, data from the arterial outlet of CPB was not included in the analysis. Moreover, blood temperature from the pulmonary artery catheter was not measured in our study. Further studies simultaneously comparing T_ET_ with T_NP_ and blood temperature might provide additional information on the discrepancies observed in this study.

## 5. Conclusions

T_ET_ recorded with a thermistor embedded between the double-layer of a balloon cuff of an endotracheal tube was a reliable alternative to T_NP_ for core body temperature measurement during steady normothermia. During rapid temperature changes, T_ET_ showed an earlier response than did T_NP_. T_ET_ could provide a reliable and accurate measurement of the core body temperature during cardiac surgery using CPB, and further studies simultaneously comparing T_ET_ with T_NP_ and blood temperature from the pulmonary artery catheter and/or arterial outlet of CPB are needed.

## Figures and Tables

**Table 1:** Pre- and intraoperative data of the participants.

Variables	Participants (*n* = 24)
Age (years)	65.5 (56.0–70.0)
Female	12 (50.0%)
Diabetes mellitus	6 (25.0%)
Hypertension	16 (66.7%)
Cerebrovascular accident	3 (12.5%)
Peripheral vascular disease	0
Chronic obstructive pulmonary disease	2 (8.3%)
Chronic kidney disease	3 (12.5%)
Renal replacement therapy	2 (8.3%)
Primary percutaneous coronary intervention	1 (4.2%)
Coronary artery bypass grafting	0
Myocardial infarction	0
Congestive heart failure	2 (8.3%)
European System for Cardiac Operative Risk Evaluation II	1.5 (0.9–2.2)
Aortic cross-clamp duration (min)	261.4 ± 77.5
Total operation time (min)	80.5 ± 36.3

Values are median (interquartile range), mean ± standard deviation, or number (%).

## Data Availability

The data used in this study are available upon reasonable request from the corresponding author, but cannot be made publicly available due to the privacy of the patients.

## Supplementary Materials

The following supporting information can be downloaded at https://www.mdpi.com/article/10.3390/jcm14020632/s1, Figure S1: Bland–Altman analysis for tracheal temperature versus nasopharyngeal temperature throughout the entire operation period; Figure S2: Concordance correlation coefficient with time lag on tracheal temperature.
