# Supplementary material for: Reliability of Tracheal Temperature as a Measurement of Core Body Temperature During Cardiac Surgery Using Cardiopulmonary Bypass
Source: J Clin Med. 2025 Jan 19;14(2):632. doi: 10.3390/jcm14020632 (PMC11765746; doi:10.3390/jcm14020632)
Supplement: Supplementary file 1 [file jcm-14-00632-s001.zip › jcm-3366593-supplementary.pdf]

# Supplementary Figures

**Figure S1.** Bland–Altman analysis for tracheal temperature versus nasopharyngeal temperature throughout the entire operation period. The horizontal dashed line (blue) indicates the mean bias. The horizontal dotted lines (red) indicate the levels of agreement. $T_{ET}$, tracheal temperature; $T_{NP}$, nasopharyngeal temperature; CI, confidence interval; LOA, limit of agreement; SD, standard deviation.

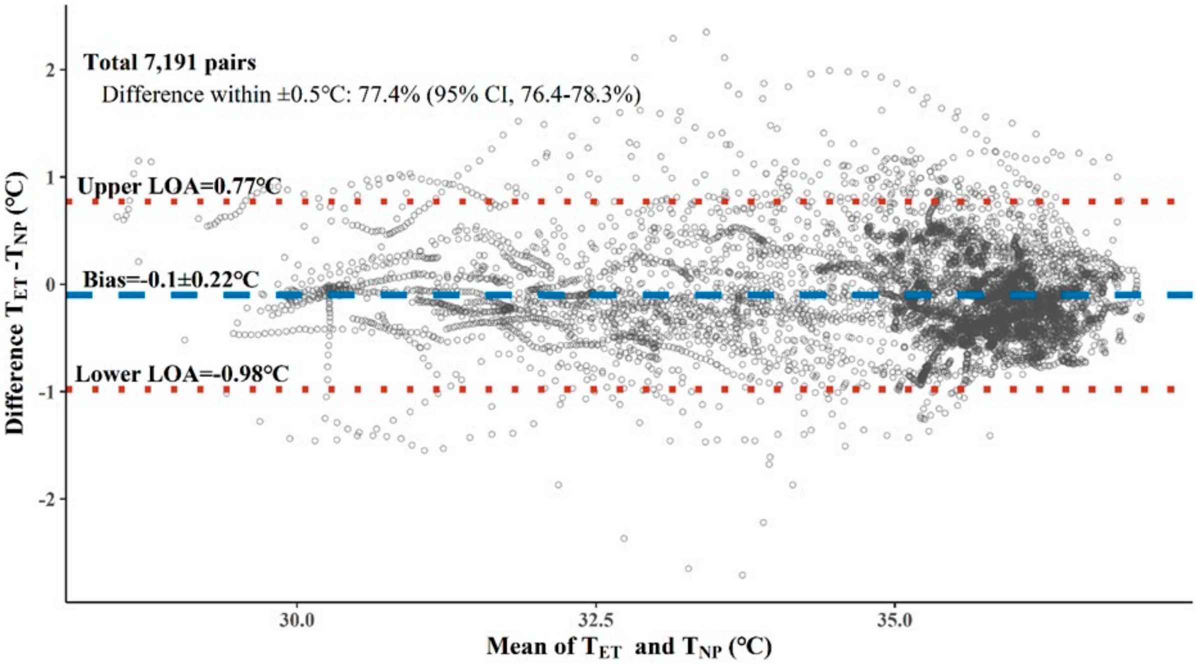

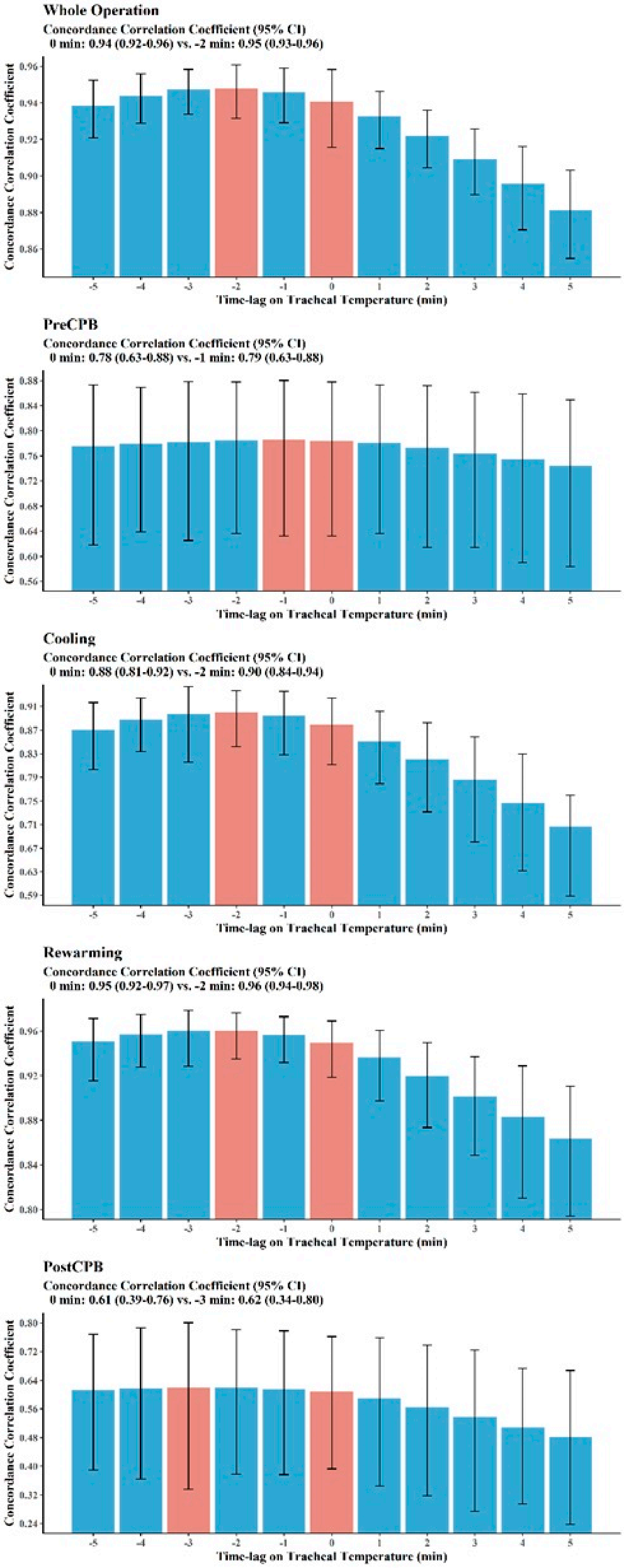
Whole Operation
Concordance Correlation Coefficient (95% CI)
0 min: 0.94 (0.92-0.96) vs. -2 min: 0.95 (0.93-0.96)
Concordance Correlation Coefficient
Time-lag on Tracheal Temperature (min)
PreCPB
Concordance Correlation Coefficient (95% CI)
0 min: 0.78 (0.63-0.88) vs. -1 min: 0.79 (0.63-0.88)
Concordance Correlation Coefficient
Time-lag on Tracheal Temperature (min)
Cooling
Concordance Correlation Coefficient (95% CI)
0 min: 0.88 (0.81-0.92) vs. -2 min: 0.90 (0.84-0.94)
Concordance Correlation Coefficient
Time-lag on Tracheal Temperature (min)
Rewarming
Concordance Correlation Coefficient (95% CI)
0 min: 0.95 (0.92-0.97) vs. -2 min: 0.96 (0.94-0.98)
Concordance Correlation Coefficient
Time-lag on Tracheal Temperature (min)
PostCPB
Concordance Correlation Coefficient (95% CI)
0 min: 0.61 (0.39-0.76) vs. -3 min: 0.62 (0.34-0.80)
Concordance Correlation Coefficient
Time-lag on Tracheal Temperature (min)

**Figure S2.** Concordance correlation coefficient with time lag on tracheal temperature. The concordance correlation coefficient without time lag (0 min) and with time lag maximizing the coefficient are distinguished by the color in each graph. CPB, cardiopulmonary bypass; CI, confidence interval; PreCPB, preparation period for the initiation of CPB; Cooling, from the initiation of CPB to the time of the detection of the lowest nasopharyngeal temperature; Rewarming, from the time of the detection of the lowest nasopharyngeal temperature to the termination of CPB; PostCPB, from the termination of CPB to the end of the operation.
